# Theta burst stimulation: what role does it play in stroke rehabilitation? A systematic review of the existing evidence

**DOI:** 10.1186/s12883-023-03492-0

**Published:** 2024-02-01

**Authors:** Tingting Jiang, Xiupan Wei, Mingzhu Wang, Jiang Xu, Nan Xia, Min Lu

**Affiliations:** Department of Rehabilitation Medicine, Tongji Hospital, Tongji Medical College, Huazhong University of Science and Technology, Wuhan, China

**Keywords:** Theta burst stimulation (TBS), Stroke, Dysfunction, Rehabilitation, Neuroplasticity

## Abstract

Various post-stroke dysfunctions often result in poor long-term outcomes for stroke survivors, but the effect of conventional treatments is limited. In recent years, lots of studies have confirmed the effect of repetitive transcranial magnetic stimulation (rTMS) in stroke rehabilitation. As a new pattern of rTMS, theta burst stimulation (TBS) was proved recently to yield more pronounced and long-lasting after-effects than the conventional pattern at a shorter stimulation duration. To explore the role of TBS in stroke rehabilitation, this review summarizes the existing evidence from all the randomized controlled trials (RCTs) so far on the efficacy of TBS applied to different post-stroke dysfunctions, including cognitive impairment, visuospatial neglect, aphasia, dysphagia, spasticity, and motor dysfunction. Overall, TBS promotes the progress of stroke rehabilitation and may serve as a preferable alternative to traditional rTMS. However, it’s hard to recommend a specific paradigm of TBS due to the limited number of current studies and their heterogeneity. Further high-quality clinical RCTs are needed to determine the optimal technical settings and intervention time in stroke survivors.

**Supplementary Information:**

The online version contains supplementary material available at 10.1186/s12883-023-03492-0.

## Introduction

Stroke is an episode of focal injury of the central nervous system (CNS) from either ischemic infarction or hemorrhage [1], constituting one of the leading reasons for acquired disability. However, most conventional therapies [2] for post-stroke dysfunction require the active participation of the patient, resulting in limited efficacy. The therapeutic potential and applications of repetitive transcranial magnetic stimulation (rTMS) [3] for facilitating post-stroke functional recovery has aroused great interest in recent years. Based on the principle of electromagnetic induction, TMS could generate subthreshold or suprathreshold currents in the cerebral cortex to regulate cortical excitability and induce neural network reorganization.

**Fig. 1:**
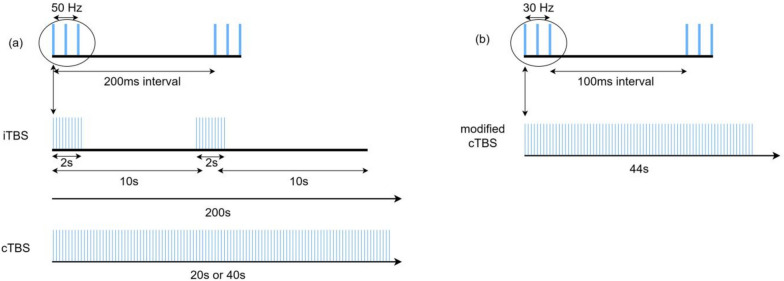
Schematic illustration of different TBS protocols

Patterned rTMS emerged during the optimization of rTMS protocol [4], which refers to the repeated use of short, high internal rate rTMS pulses interspersed with short pauses of no stimulation. By far the most commonly used protocol is theta burst stimulation (TBS) [5], which has presented advantages over other conventional rTMS strategies in its low intensity, short duration of application, and long-lasting effects [6, 7]. In particular, the TBS protocol has been used to mimic the brain’s natural firing patterns to upregulate or downregulate the excitability of focal regions of the cortical surface with relatively high accuracy [8]. The basic element of TBS is a burst of 3 pulses at a frequency of 50 Hz every 200 ms (Fig. 1a). Two main patterns are commonly used, intermittent TBS (iTBS) and continuous TBS (cTBS). In iTBS, 10 short sequences of 2 s duration are given every 10 s for 20 cycles, associated with excitatory after-effects of cortical activity, whereas in cTBS 100 or 200 bursts are given in succession for 20 s or 40 s to show the inhibitory after-effects. Besides, some studies [9–13] have used a modified cTBS protocol with a total of 801 pulses (each burst consisting of 3 pulses at 30 Hz, repeated at 6 Hz) and lasting for 44 s (Fig. 1b).

The interhemispheric inhibition (IHI) model [14] forms the basis of most studies using TBS as a treatment tool in stroke rehabilitation. Stroke disrupts the inhibitional balance between the hemispheres causing ipsilateral damage coupled with excess inhibition from the opposite hemisphere, the imbalance can be normalized by (cTBS) suppressing the excitability of the unaffected hemisphere or (iTBS) upregulating the excitability of the ipsilesional hemisphere.

Although studies on TBS intervention for specific post-stroke dysfunctions have been published [15, 16], there is still a lack of conclusive statements on the role of TBS in stroke rehabilitation. This review aims to summarize the current evidence of TBS in the rehabilitation of various post-stroke dysfunctions, providing directions for clinical application and future research using TBS to promote stroke rehabilitation.

## Method

The study protocol was registered on the International Prospective Register of Systematic Reviews (PROSPERO, CRD42023460336). Furthermore, our review was conducted based on the Preferred Reporting Items for Systematic Reviews and Meta-analyses (PRISMA) statement.

### Search strategy

PubMed, Ovid Medline, Cochrane library, Embase, and Web of Science were searched for all the randomized controlled trials (RCTs) on TBS protocols in post-stroke rehabilitation, published in English up to August 2023 by using the following search terms including “theta burst stimulation”, "TBS”, “cerebrovascular accident” and “Stroke”. Reference lists of identified RCTs and other systematic reviews and meta-analyses were also manually searched to identify additional studies. We focused on the recovery of cognitive impairment, visuospatial neglect, aphasia, dysphagia, spasticity, and motor dysfunction. All animal experiments were ruled out. The search strategy is illustrated in Supplementary file 1.

### Eligibility criteria

The inclusion criteria of this study were based on the five main principles of the Participant-Intervention-Comparator-Outcomes-Study design (PICOS): (1) Population: patients with different dysfunctions after stroke. (2) Intervention: cTBS or iTBS, excluding combined other NIBS techniques (conventional rTMS and tDCS). (3) Comparison: sham stimulation or no stimulation. (4) Outcome: various post-stroke dysfunctions, including cognitive impairment, visuospatial neglect, aphasia, dysphagia, spasticity, and motor dysfunction. As various measures were used in these studies, the outcomes related to specific dysfunction that were used in more than two studies were selected for the Meta-analysis. Measurements available for meta-analysis include line bisection test (LBT) and star cancellation test (SCT) for visuospatial neglect, Modified Ashworth scale (MAS) for spasticity, Fugl-Meyer Assessment (FMA), Action Research Arm Test (ARAT), Nine-hole Peg Test (NHPT) and Berg Balance Scale (BBS) for motor dysfunction. (5) Study design: RCTs (excluding cross-over studies); Each group consisted of a minimum of five participants; Regular rehabilitation training was allowed whether utilizing TBS or not.

The following studies were excluded: reviews or commentaries, basic experiments, a summary of meetings, book chapters, case reports, full text is not available, unpublished, or duplicate literature.

### Study selection and data extraction

First, two independent reviewers (MW and XW) completed the search and identification of eligible studies. All duplicate documents were removed by using EndNote X8. Then, the titles and abstracts were read to select papers that met the criteria. The full text of all relevant studies was subsequently retrieved and further examined carefully. Any disagreements were resolved by discussion with a third senior reviewer (ML).

Data extraction was conducted independently by TJ and JX using Microsoft Office Excel. The following variables were extracted from studies: (1) the general characteristics including authors, year of publication; (2) sample characteristics including sample size, age, side of the lesion, type of stroke, and course of disease; (3) interventions and control protocols, intervention period, targeted area, adjuvant therapy; (4) outcomes; (5) follow-up (6) adverse effects. The mean scores and standard deviations (SD) of the outcomes at baseline and post-intervention were extracted, as well as the mean change scores and SD for meta-analyses. If there were several groups in the included RCTs, only those that were congruent with the systematic review's aims would be extracted. If no numerical data were provided, we contacted the authors or extracted the data from the figures using Web-Plot-Digitizer. If the standard error of the mean (SEM) was provided, it was converted to SD by using the formula of SD = SEM × √n. For some studies in which the change of SD from baseline to endpoint is not given, we roughly estimate the SD value by calculating the correlation coefficients.

### Methodological quality assessment

The quality of all included RCTs was evaluated independently by two authors (TJ and JX), according to the Cochrane Collaboration's tool. The assessment included random sequence generation, allocation concealment, blinding of patients and study personnel, blinding of outcome assessment, completeness of outcome data, selective reporting of outcomes, and other possible biases. The risk of bias can be divided into high, uncertain, or low. Discrepancies in the assessment were resolved through discussion until a consensus was reached. We planned to assess the potential publication bias by funnel plots, but every meta-analysis contained fewer than 10 studies when sorted by outcomes, in which case the funnel plots could yield misleading results and are not recommended.

### Statistical analysis

Meta-analyses were performed using the Review Manager software (RevMan, version 5.4). The effects and corresponding 95% confidence intervals (CIs) were used to compare the outcomes. The outcome indicators included in the study are all continuous variables, the weighted mean difference (WMD) or standard mean difference (SMD) were used to represent the magnitude of the effect. The I^2^ statistic and Cochrane's Q test were applied to evaluate heterogeneity among the included studies. I^2^ > 50% and *P* < 0.10 to the Q test indicated high heterogeneity, and the random-effects model was used; otherwise, the fixed-effects model was applied. *P* < 0.05 was considered statistically different. In the case of considerable heterogeneity (i.e. I^2^ > 75%), we would have conducted subgroup analyses and sensitivity analyses to identify the sources of heterogeneity. If there were two or fewer studies identified for a single analysis objective, we would not perform a meta-analysis but provide a narrative synthesis of the findings only. We planned possible subgroup analyses according to the following characteristics:

(1) Type of stimulation: iTBS vs cTBS; (2) Number of cTBS pulses: 600 pulses vs 801 pulses vs 1200 pulses; (3) Follow-up: short-term (≤ 1 month) vs long-term (> 1 month); (4) Course of disease: acute/subacute phase vs chronic phase; (5) Targeted area. Furthermore, we planned a sensitivity analysis on the methodological quality of studies by excluding studies with a high risk of bias.

## Results

### Study selection and characteristics

**Fig. 2:**
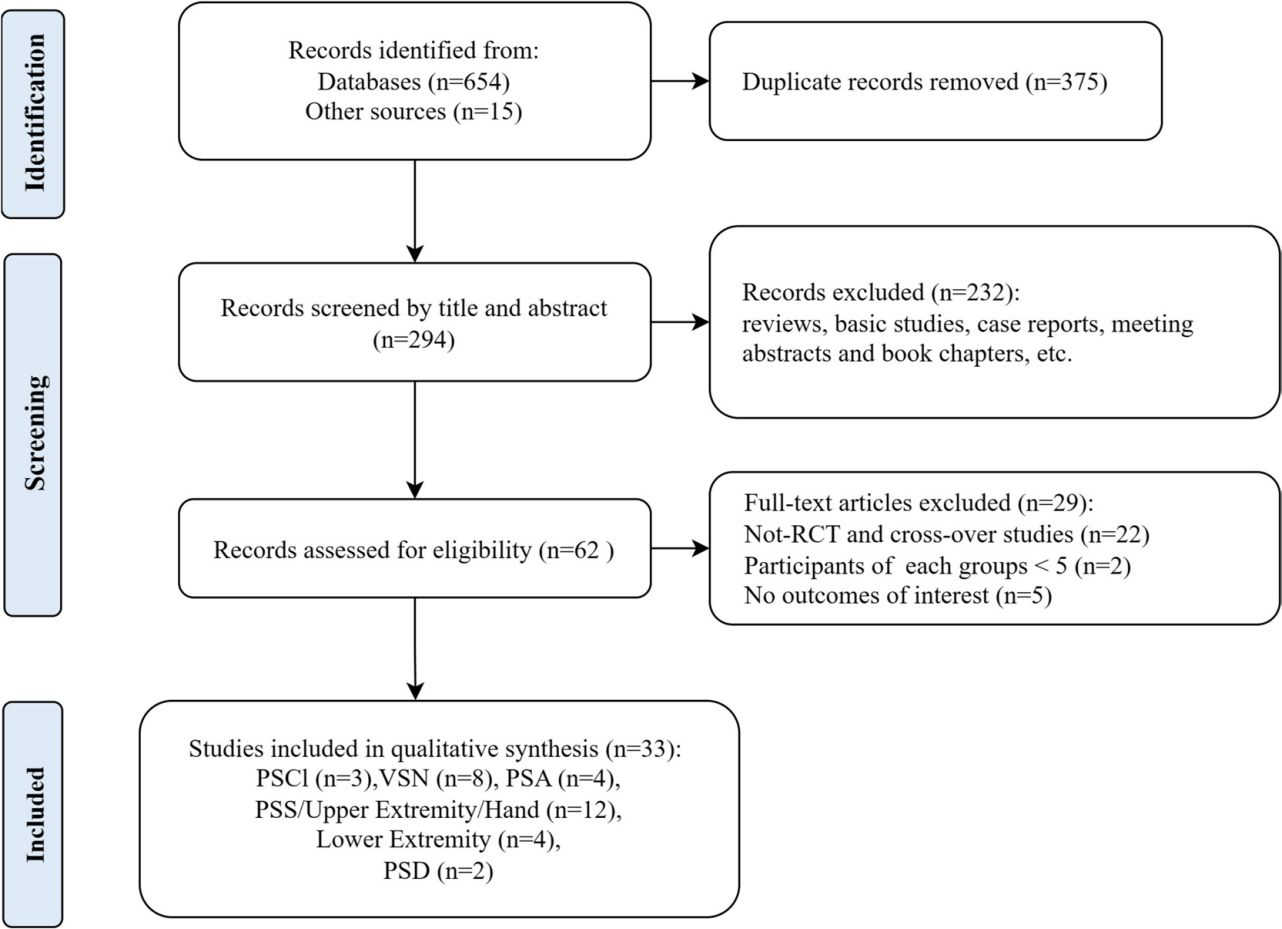
Flow chart presenting the selection of eligible studies

The literature search process is presented in Fig. 2. A total of 33 published RCTs using TBS in stroke rehabilitation have reported results and carried out the corresponding analysis. In detail, the results included the following: [17–19] three for cognitive impairment, [9–13, 20–22] eight for visuospatial neglect, [23–26] four for aphasia, [27–38] twelve for spasticity and upper extremity/hand motor dysfunction, [39–42] four for lower extremity/balance and [43, 44] two for dysphagia.

Clinical and demographical features of the included studies are provided in Table 1. The number of participants in each study ranged from 12 [22, 37] to 64 [44]. Most participants of those reported were male (65.49%) with a mean (SD) age ranging from 48.78 (11.34) [25] to 75.20 (5.50) [31] years. The onset time of all participants included in the study may be as short as 1 week [31] or as long as 6 months or more. Thirty out of the 33 included studies reported using TBS combined with other conventional rehabilitation therapies or medical treatments. As regards the stimulation pattern of TBS, the majority of studies gave 600 pulses total for TBS intervention with an intensity ranging from 60% resting motor threshold (RMT) [35] to 110% RMT [31] or from 80% active motor threshold (AMT) to 100% AMT [10], except for one gave 1,200 pulses in total [41] and five gave 801 pulses in total [9–13]. The duration of treatment ranges from 1 to 4 sessions per day. The course of treatment ranged from 10 days to 6 weeks. The control group in most studies used sham TBS, except for two used an intensity of 40% RMT [20, 22] and one used no stimulation [35].

**Table 1:** Main characteristics of studies included in the systematic review (*n* = 33)

Clinical feature	Group	Sample size(Male/Female)	Age (years)	Side(Left/Right)	Type (Ischemic/ Hemorrhagic)	Course of disease	TBS protocol	Targeted Area	Outcomes	Follow-up	Adjuvant therapy	Adverse effects (n)
**Cognitive impairment**												
Li et al.[18]	iTBS	16/12	69.50 ± 9.50	16/12	18/10	25.00 d	600 pulses, 100% RMT, 1 sess/d, 2 weeks	LH DLPFC	MMSE, OCS	/	Conventional rehabilitation therapies	sneezing: 1
	sham	18/12	66.00 ± 13.00	24/6	14/16	25.00 d						/
Chu et al.[19]	iTBS	18/3	57.24 ± 14.03	12/9	13/8	4.00 ± 5.00 mon	600 pulses, 70% RMT, 1 sess/d, 6 weeks	LH DLPFC	MMSE, LOTCA,	/	Computer- assisted rehabilitation therapy	/
	sham	13/7	66.75 ± 12.23	13/7	12/8	6.00 ± 4.00 mon						
Tsai et al.[17]	iTBS	11/4	60.13 ± 14.10	Left	7/8	18.47 ± 20.21 d	600 pulses, 80% RMT, 1 sess/d, 2 weeks	LH DLPFC	RBANS	/	N/A	/
	sham	13/2	56.23 ± 12.00		10/5	38.00 ± 7.90 d						
**Visuospatial neglect**												
Koch et al.[21]	cTBS	N/A	61.40 ± 13.02	Right	Ischemic	46.80 d	600 pulses, 80% AMT, 2 sess/d, 2 weeks	LH PPC	BIT	2 weeks	Computer- assisted rehabilitation therapy	/
	sham	N/A	71.89 ± 4.86			36.90 d						
Fu et al.[11]	cTBS	8/2	55.10 ± 13.96	Right	5/5	43.70 ± 23.50 d	801 pulses, 80% RMT, 4 sess/d, 14 days	LH PPC	SCT, LBT	4 weeks	Conventional rehabilitation therapies	slight dizziness: 2
	sham	8/2	59.50 ± 12.68			59.50 ± 12.00 d						/
Yang et al.[9]	cTBS	5/4	49.45 ± 10.78	N/A	6/3	104.85 ± 36.38 d	801 pulses, 80% RMT, 4 sess/d, 2 weeks	LH PPC	SCT, LBT	1 mon	Conventional rehabilitation therapies	N/A
	sham	3/7	47.70 ± 11.81		6/4	105.91 ± 37.59 d						
Fu et al.[22]	cTBS	9/3	60.17 ± 14.05	Right	N/A	41.83 ± 20.56 d	600 pulses, 80% RMT, 4sess/day, 10 days	LH PPC	SCT, LBT	/	Conventional rehabilitation therapies	/
	control		62.00 ± 9.78		N/A	36.17 ± 17.50 d	600 pulses, 40% RMT, 4 sess/day, 10 days					
Cao et al.[20]	iTBS	6/1	55.00 ± 12.00	Right	N/A	32.00 ± 17.00 d	600 pulses, 80% RMT, 2 sess/day, 2 weeks	LH DLPFC	SCT, LBT	/	Conventional rehabilitation therapies	N/A
	control	5/1	62.00 ± 10.00		N/A	36.00 ± 17.00 d	600 pulses, 40% RMT, 2 sess/day, 2 weeks					
Nyffeler et al.[13]	8cTBS	5/5	67.80 ± 10.13	Right	N/A	26.80 ± 20.89 d	801 pulses, 100% RMT, 4sess/day, 2 days	LH PPC	CBS	3 mons	Conventional rehabilitation therapies	/
	16cTBS	6/4	74.30 ± 10.23		N/A	22.90 ± 10.34 d	801 pulses, 100% RMT, 4 sess/day, 4 days					
	sham	7/3	70.60 ± 11.44		N/A	25.80 ± 11.26 d	801 pulses, 100% RMT, 4sess/day, 2 days					
Hopfner et al.[10]	cTBS	5/7	62.00 ± 10.60	Right	N/A	32.42 ± 11.95 d	801 pulses, 100% AMT, 2 sess/day, 2 weeks	LH PPC	BCT	/	Smooth pursuit training	N/A
	sham	4/2	65.40 ± 15.20		N/A	32.42 ± 11.95 d						
Vatanparasti et al.[12]	cTBS	5/2	67.50 ± 8.40	N/A	4/3	N/A	801 pulses, 80% RMT, 1 sess/day, 2 weeks	LH PPC	SCT, LBT	/	Prism adaptation	/
	sham	5/2	65.50 ± 10.20		4/3	N/A						
**Aphasia**												
Chou et al.[26]	iTBS	15/14	62.70 ± 12.70	Left	20/9	17.60 ± 20.80 mon	600 pulses, 80% RMT, 1 sess/day, 2 weeks	LH IFG	CCAT	/	Speech and language therapy	/
	sham	20/9	61.60 ± 14.70		20/9	16.50 ± 24.60 mon						
Allendorfer et al.[24]	iTBS	12/6	59.61 ± 10.54	Left	Ischemic	4.62 ± 4.17 y	600 pulses, 80% AMT, 1 sess/day, 3 weeks	LH IFG	BNT,SFT,COWAT, WAB-R AQ,PPVT,	3 mons	N/A	N/A
	Sham	4/2	43.78 ± 13.98			3.94 ± 3.56 y						
Szaflarski et al.[25]	iTBS	14/7	59.39 ± 13.83	Left	Ischemic	3.90 ± 4.02 y	600 pulses, 80% AMT, 1 sess/day, 3 weeks	LH IFG	BNT,SFT,COWAT, WAB	3 mons	N/A	N/A
	sham	4/2	48.78 ± 11.34			4.62 ± 4.02 y						
Zheng et al.[23]	cTBS	13/2	53.13 ± 11.26	Left	N/A	42.80 ± 12.91 d	600 pulses, 80% AMT, 1 sess/day, 3 weeks	RH pSTG	WAB-AQ	/	Speech and language therapy	N/A
	Sham	11/3	66.33 ± 13.77		N/A	50.50 ± 21.93 d						
**Spasticity /Upper Extremity** **/Hand**												
Chen et al.[29]	iTBS	7/4	52.90 ± 11.10	6/5	2/9	> 6 mon	600 pulse, 80% AMT, 1 sess/day, 2 weeks	AH M1	MAS, FMA-UE, ARAT	/	Physical and occupational therapy	/
	sham	7/4	52.60 ± 8.30	9/2	3/8							
Kuzu et al.[27]	cTBS	6/1	61.30 ± 9.80	2/5	Ischemic	14.50 ± 1.60 mon	600 pulse, 80% AMT, 1 sess/day, 2 weeks	UH M1	MAS, FMA-UE	4 weeks	Conventional rehabilitation therapies	/
	sham	2/4	65.00 ± 4.60	3/3		14.50 ± 2.00 mon						
Chen et al.[28]	iTBS	13/3	57.38 ± 8.04	4/12	10/6	80.13 ± 35.19 d	600 pulse, 80% AMT, 1 sess/day, 2 weeks	Ipsilesional CB	MAS	/	Physical therapy	/
	sham	12/4	51.44 ± 9.19	9/7	8/8	101.50 ± 54.15 d						
Watanabe et al.[31]	iTBS	5/3	72.50 ± 6.50	4/4	N/A	< 1 week	600 pulses, 110%RMT, 1 sess /day, 10 days	AH M1	FMA-UE, MAS	/	Conventional rehabilitation therapies	/
	sham	3/3	75.20 ± 5.50	1/5	N/A							
Ackerley et al.[32]	iTBS	6/3	61	3/6	N/A	20 mon	600 pulses, 90% AMT, 1 sess /day, 10 days	AH M1	FMA-UE, ARAT	1 mon and 3 mons	Conventional rehabilitation therapies	/
	sham	6/3	71	3/6	N/A	18 mon						
Chen et al.[33]	iTBS	8/4	54.36 ± 10.56	7/5	6/6	5.01 ± 4.39 mon	600 pulses, 80% AMT, 2 sess/day, 15 days	AH M1	MAS, FMA-UE, ARAT, NHPT, BBT,	/	Virtual reality-based cycling training	/
	sham	10/1	48.95 ± 9.63	7/4	2/9	7.99 ± 5.41 mon						
Talelli et al.[38]	iTBS	7/6	54.4 ± 15.8	9/4	Ischemic	17.50 ± 5.10 mon	600 pulses, 80% AMT, 1 sess /day; 2 weeks	AH M1	NHPT, JTT, grip strength	1 mon and 3 mons	Physical therapy	/
	sham	9/3	58.5 ± 12.0	5/7		38.50 ± 57.20 mon						
	cTBS	7/5	55.8 ± 12.4	2/10		29.80 ± 19.70 mon	600 pulses, 80% AMT, 1 sess /day, 2 weeks	UH M1				
	sham	6/6	59.4 ± 12.4	7/5		49.60 ± 76.90 mon						
Sung et al.[34]	iTBS	9/3	64.20 ± 11.90	N/A	8/4	8.10 ± 1.5 mon	600 pulses, 80% AMT, 10 sess/day, 4 weeks	AH M1	FMA-UE, WMFT	/	Physical therapy and occupational therapy	/
	sham	11/3	63.10 ± 12.80	N/A	9/5	8.20 ± 1.60 mon						
Khan et al.[35]	TBS	13/7	63.55 ± 12.67	6/14	Ischemic	17.10 ± 4.82 d	600 pulses, 60%RMT, 1 sess /day, 4 weeks	iTBS to AH M1 + cTBS to UH M1	FMA-UE	3 mon, 6 mon and 1 y	Physical therapy	N/A
	no sti	12/8	64.60 ± 12.99	5/15		16.40 ± 5.58 d						
Di Lazzaro et al.[37]	cTBS	3/3	59.5 ± 12.4	1/5	Ischemic	34.80 ± 17.50 mon	600 pulses, 80% AMT, 1 sess /day, 10 days	AH M1	ARAT, NHPT, JTT	1 mon and 3 mons	Physical therapy	/
	sham	4/2	57.5 ± 12.3	2/4		30.00 ± 27.60 mon						
Zhang et al.[36]	cTBS + iTBS	9/5	58.21 ± 9.00	8/6	6/8	50.57 ± 32.01 mon	600 pulses, 70% RMT, 1 sess /day, 3 weeks	AH M1	FMA-UE, ARAT	2 weeks	Robot- assisted training	N/A
	sham cTBS + iTBS	7/7	59.50 ± 8.56	8/6	8/6	63.93 ± 46.80 mon						
	sham cTBS + sham iTBS	8/6	64.00 ± 5.39	7/7	10/4	50.86 ± 29.50 mon						
Nicolo et al.[30]	cTBS [14]	7/7	62.40 ± 12.30	4/10	13/1	5.30 ± 1.80 weeks	600 pulses, 70% RMT, 3 sess/week, 3 weeks	UH M1	FMA-UE, NHPT	1 mon	Physical therapy	/
	sham [13]	8/5	64.30 ± 17.10	3/10	10/3	4.70 ± 1.40 weeks						
**Lower Extremity /Balance**												
Koch et al.[40]	iTBS	13/4	64.00 ± 11.30	6/11	Ischemic	15.18 ± 6.63 mon	600 pulse, 80% AMT, 2 sess/day, 3 weeks	ipsilesional CB	BBS, FMA-LE	3 weeks	Physical therapy	/
	sham	10/7		8/9		15.88 ± 6.00 mon						
Liao et al.[42]	iTBS	12/3	51.53 ± 9.22	11/4	7/8	70.40 ± 44.43 d	600 pulse, 80% AMT, 1 sess/day, 2 weeks	ipsilesional CB	BBS, FMA-LE	/	Physical therapy	slight headache: 1
	sham	9/6	55.40 ± 8.10	9/6	8/7	86.53 ± 45.26 d						/
Lin et al.[41]	iTBS	1/9	60.80 ± 8.10	5/5	7/3	359.00 ± 171.00 d	1200 pulses, 100% midline MT, 2 sess/week, 5 weeks	Bilateral M1	BBS, FMA-LE	/	Conventional rehabilitation therapies	/
	sham	2/8	61.10 ± 9.70	6/4	9/1	384.00 ± 270.00 d						
Xie et al.[39]	iTBS	13/5	52.35 ± 8.62	7/11	10/8	2.22 ± 1.70 mon	600 pulse, 80% AMT, 1 sess/day, 2 weeks	contralesional CB	FMA-LE	/	Conventional rehabilitation therapies	/
	sham	11/7	54.41 ± 7.01	6/12	10/8	2.91 ± 1.96 mon						
**Dysphagia**												
Xie et al.[43]	iTBS	12/6	66.90 ± 11.30	9/9	14/4	21.10 ± 20.11 d	600 pulses, 80% RMT, 1 sess/day, 2 weeks	Affected suprahyoid motor cortex	SSA, WST, MSS, PAS	2 weeks	Conventional swallowing therapy	/
	sham	14/6	62.80 ± 13.00	8/12	13/7	23.70 ± 16.00 d						
Rao et al.[44]	iTBS	22/11	63.42 ± 10.35	14/18	15/18	22.00 ± 8.00 d	600 pulses, 100% RMT, 1 sess/day, 2 weeks	Bilateral CB	FEDSS, PAS,SSA, FOIS	4 weeks	Conventional swallowing therapy	slight dizziness: 3
	sham	24/7	65.90 ± 11.42	12/12	19/12	26.00 ± 3.00 d						/

*Abbreviations: AMT* Active motor threshold, *AH* Affcted hemisphere, *AQ* Aphasia Quotient, *ARAT* Action Research Arm Test, *BBS* Berg Balance Scale, *BBT* Box and Block test, *BCT* Bird Cancellation Task, *BIT* Behavioral Inattention Test, *BNT* Boston Naming Test, *CB* Cerebellum, *CBS* Catherine Bergego Scale, *CCAT* Concise Chinese Aphasia Test, *COWAT* Controlled Oral Word Association Test, *DLPFC* Dorsolateral prefrontal cortex, *FEDSS* Fiberoptic Endoscopic Dysphagia Severity Scale, *FMA* Fugl-Meyer Assessment, *FOIS* Functional Oral Intake Scale, *IFG* Inferior frontal gyrus, *iTBS* intermittent theta burst stimulation, *JTT* Jebsen Taylor Test, *LBT* Line bisection test, *LE* Lower extremity, *LH* Left hemisphere, *LOTCA* Loewenstein Occupational Therapy Cognitive Assessment, *MAS* Modified Ashworth scale, *M1* Primary motor cortex, *MMSE* Mini‐mental state examination, *MSS* Murray Secretion Scale, *NHPT* Nine-hole peg test, *N/A* Not available, *OCS* Oxford cognitive screen, *PAS* Penetration/Aspiration Scale, *PPC* Posterior parietal cortex, *PPVT* Peabody Picture Vocabulary Test, *pSTG* Posterior superior temporal gyrus, *RH* Right hemisphere, *RBANS* Repeatable Battery for the Assessment of Neuropsychological Status, *RMT* Resting motor threshold, *SCT* Star cancellation test, *SFT* Semantic Fluency Test, *SSA* Standardized Swallowing Assessment, *UE* Upper extremity, *UH* Unaffcted hemisphere, *WAB-R* Western Aphasia Battery-Revised, *WMFT* Wolf Motor Function Test, *WST* Water-swallowing test

Among 33 included studies, twenty-one reported no obvious adverse effects. Four studies reported minor adverse effects [11, 18, 42, 44] in TBS group, such as sneezing, slight headache or dizziness, which were tolerable, and the experiment could be continued.

### Quality assessment

**Fig. 3:**
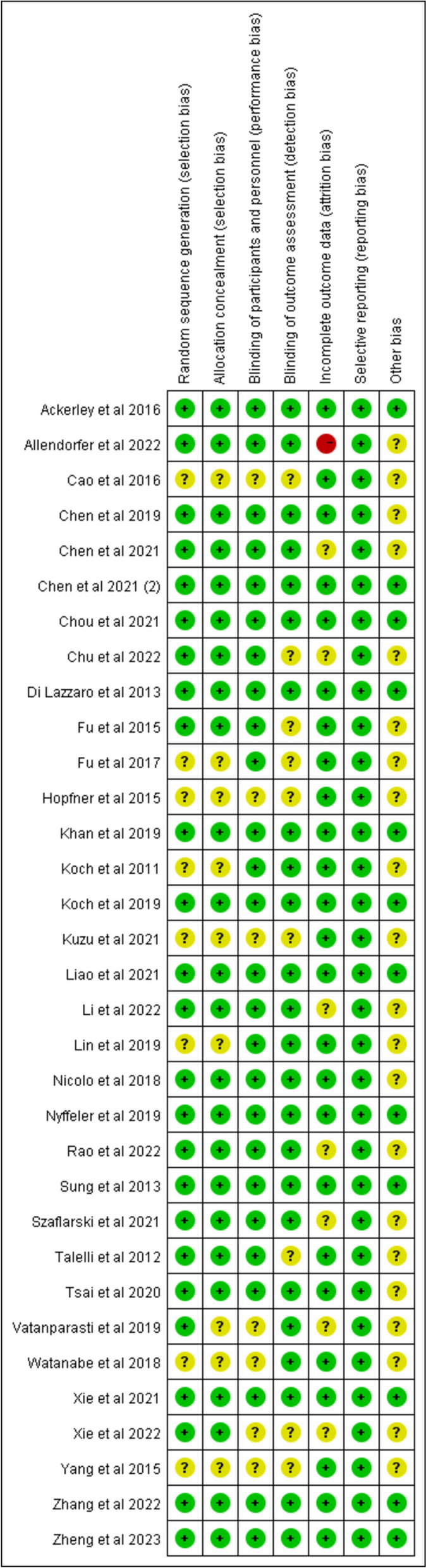
Risk of bias summary

 \* MERGEFORMAT Fig. 3 presents the details of the risk bias for all included studies. Nine studies had an unclear risk of bias in random sequence generation and allocation schemes. For blinding of participants and personnel, the risk of bias was unclear in six studies. Six studies had a low risk related to blinding of outcome assessment. For incomplete outcome data, six studies exhibited a low risk and one study exhibited a high risk. All presented a low risk for selective reporting. Therefore, all of the included studies presented moderate to high methodological quality.

### Effects of interventions

We conducted meta-analyses and further subgroup analyses on studies with three or more common outcome measures to determine potential TBS effects. Results were assessed for their quality of evidence using www.gradepro.org, and for studies that implemented sensitivity analyses, only the results with reduced heterogeneity were evaluated. All conclusions are presented in Table 2.

**Table 2:** Subgroup analysis and quality of the evidence

	**Studies (n)**	**WMD**/**SMD [95%CI]**	***P***	**I**^**2**^** (%)**	***P (heterogeneity)***	**Certainty of the evidence**		
						**No of participants (studies)**	**GRADE**	**Consideration**
**LBT**	6	-1.11 [-2.04, -0.17]	0.02	74	0.002	96 (6 studies)	**⨁⨁◯◯ low**	Due to risk of bias and inconsistency
**Type of stimulation**								
iTBS	1	-3.75 [-5.80, -1.71]	0.0003	N/A	N/A	13 (1 studies)	**⨁⨁◯◯ low**	Due to risk of bias and imprecision
cTBS	5	-0.71 [-1.48, 0.06]	0.07	62	0.03	83 (5 studies)	**⨁⨁◯◯ low**	Due to risk of bias and inconsistency
**Number of TBS pulses**								
600 pulses	3	-2.08 [-4.50, 0.34]	0.09	86	0.0006	43 (3 studies)	**⨁**◯◯◯ **very low**	Due to risk of bias and inconsistency
801 pulses	3	-0.54 [-1.26, 0.18]	0.14	39	0.2	53 (3 studies)	**⨁⨁⨁**◯** moderate**	Due to risk of bias
**Follow-up**								
short-term (≤ 1 month)	3	-1.02 [-1.98, -0.05]	0.04	64	0.06	57 (3 studies)	**⨁⨁◯◯ low**	Due to risk of bias and inconsistency
long-term (> 1 month)	/							
**SCT**	6	-2.31 [-3.80, -0.81]	0.002	85	< 0.00001	96 (6 studies)	**⨁**◯◯◯** very low**	Due to risk of bias and inconsistency
**Type of stimulation**								
iTBS	1	-6.67 [-9.93, -3.41]	< 0.0001	N/A	N/A	13 (1 studies)	**⨁⨁⨁◯ moderate**	Due to risk of bias and imprecision
cTBS	5	-1.75 [-3.12, -0.39]	0.01	83	< 0.0001	83 (5 studies)	**⨁**◯◯◯** very low**	Due to risk of bias and inconsistency
**Number of TBS pulses**								
600 pulses	3	-3.32 [-7.28, 0.64]	0.1	91	< 0.0001	43 (3 studies)	**⨁◯◯◯ very low**	Due to risk of bias and inconsistency
801 pulses	3	-1.84 [-3.36, -0.33]	0.02	79	0.008	53 (3 studies)	**⨁◯◯◯ very low**	Due to risk of bias and inconsistency
**Follow-up**								
short-term (≤ 1 month)	3	-1.88 [-4.05, 0.29]	0.09	90	< 0.0001	39 (2 studies)	**⨁⨁⨁◯ moderate**	Due to risk of bias
long-term (> 1 month)	/							
**MAS**	5	-0.44 [-0.77, -0.12]	0.007	0	0.81	104 (5 studies)	**⨁⨁⨁◯ moderate**	Due to risk of bias
**Type of stimulation**								
iTBS	4	-0.40 [-0.75, -0.04]	0.03	0	0.77	91 (4 studies)	**⨁⨁⨁◯ moderate**	Due to risk of bias
cTBS	1	-0.70 [-1.52, 0.12]	0.09	N/A	N/A	13 (1 studies)	**⨁◯◯◯ very low**	Due to risk of bias and imprecision
**Course of disease**								
acute/subacute phase	3	-0.37 [-0.73, -0.01]	0.05	0	0.68	69 (3 studies)	**⨁⨁⨁◯ moderate**	Due to risk of bias
chronic phase	2	-0.73 [-1.45, -0.02]	0.05	0	0.87	35 (2 studies)	**⨁⨁⨁◯ moderate**	Due to risk of bias
**Targeted area**								
Affected M1	2	-0.34 [-0.79, 0.11]	0.14	0	0.61	59 (2 studies)	**⨁⨁⨁◯ moderate**	Due to risk of bias
Ipsilesional CB	1	-0.48 [-1.04, 0.08]	0.09	N/A	N/A	32 (1 studies)	**⨁⨁⨁⨁ high**	
**NHPT**	5	-0.01 [-0.44, 0.43]	0.97	40	0.17	111 (5 studies)	**⨁⨁⨁⨁ high**	
**Type of stimulation**								
cTBS	3	-0.38 [-1.06, -0.30]	0.27	67	0.08	63 (3 studies)	**⨁⨁◯◯ low**	Due to risk of bias and inconsistency
iTBS	2	0.25 [-0.32, 0.82]	0.38	0	0.94	48 (2 studies)	**⨁⨁⨁⨁ high**	
**Course of disease**								
acute/subacute phase	2	0.27 [-0.55, 1.10]	0.51	N/A	N/A	50 (2 studies)	**⨁⨁⨁⨁ high**	
chronic phase	3	-0.12 [-0.55, 1.10]	0.65	54	0.11	61 (3 studies)	**⨁⨁⨁◯ moderate**	Due to inconsistency
**Targeted area**								
Unaffected M1	2	-0.03 [-0.06, -0.00]	0.04	N/A	N/A	51 (2 studies)	**⨁⨁⨁⨁ high**	
Affected M1	1	0.02 [-0.03, 0.07]	0.40	N/A	N/A	12 (1 studies)	**⨁⨁⨁◯ moderate**	Due to imprecision
**Follow-up**								
short-term (≤ 1 month)	4	0.00 [-0.02, 0.02]	0.83	0	0.95	87 (4 studies)	**⨁⨁⨁⨁ high**	
long-term (> 1 month)	3	-0.00 [-0.03, 0.03]	0.81	0	0.48	55 (3 studies)	**⨁◯◯◯ very low**	Due to risk of bias and inconsistency
**FMA-UE**	9	3.89 [0.60, 7.18]	0.02	88	< 0.00001	181 (8 studies)	**⨁⨁⨁⨁ high**	
**Type of stimulation**								
iTBS	6	2.03 [0.78, 3.28]	0.001	0	0.88	126 (6 studies)	**⨁⨁⨁◯ moderate**	Due to risk of bias
priming iTBS	1	3.00 [1.01, 4.99]	0.003	N/A	N/A	28 (1 studies)	**⨁⨁⨁⨁ high**	
cTBS	1	4.80 [-6.48, 16.08]	0.4	N/A	N/A	27 (1 studies)	**⨁⨁⨁⨁ high**	
TBS	1	10.00 [8.43, 11.57]	< 0.00001	N/A	N/A	40 (1studies)	**⨁⨁⨁⨁ high**	
**Course of disease**								
acute/subacute phase	3	4.28 [-1.57, 10.12]	0.15	0	0.55	64 (3 studies)	**⨁⨁⨁⨁ high**	
chronic phase	5	2.26 [1.19, 3.34]	< 0.0001	0	0.92	117 (5 studies)	**⨁⨁⨁◯ moderate**	Due to risk of bias
**Follow-up**								
short-term (≤ 1 month)	4	1.93 [0.78, 3.08]	0.001	0	0.42	96 (4 studies)	**⨁⨁⨁◯ moderate**	Due to risk of bias
long-term (> 1 month)	2	10.41 [5.71, 15.12]	< 0.0001	45	0.18	54 (2 studies)	**⨁⨁⨁⨁ high**	
**ARAT**	6	3.35 [2.78, 3.91]	< 0.00001	35	0.17	131 (6 studies)	**⨁⨁⨁⨁ high**	
**Type of stimulation**								
iTBS	4	4.11 [3.32, 4.89]	< 0.00001	0	0.98	91 (4 studies)	**⨁⨁⨁⨁ high**	
priming iTBS	1	2.56 [1.75, 3.37]	< 0.00001	N/A	N/A	28 (1 studies)	**⨁⨁⨁⨁ high**	
cTBS	1	0.35 [-11.00, 11.70]	0.95	N/A	N/A	12 (1 studies)	**⨁⨁⨁◯ moderate**	Due to imprecision
**Course of disease**								
acute/subacute phase	1	3.49 [-13.32, 20.30]	0.68	N/A	N/A	23 (1 studies)	**⨁⨁⨁⨁ high**	
chronic phase	5	3.35 [2.78, 3.91]	< 0.00001	48	0.1	108 (5 studies)	**⨁⨁⨁⨁ high**	
**Follow-up**								
short-term (≤ 1 month)	2	0.95 [-8.95, 10.85]	0.85	0	0.92	30 (2 studies)	**⨁⨁⨁⨁ high**	
long-term (> 1 month)	2	0.15 [-10.01, 10.30]	0.98	0	0.88	30 (2 studies)	**⨁⨁⨁⨁ high**	
**FMA-LE**	4	0.44 [-0.76, 1.64]	0.47	2	0.38	120 (4 studies)	**⨁⨁⨁◯ moderate**	Due to risk of bias
**Number of TBS pulses**								
600 pulses	3	1.39 [-0.94, 3.72]	0.24	8	0.34	100 (3 studies)	**⨁⨁⨁⨁ high**	
1200 pulses	1	0.10 [-1.29, 1.49]	0.89	N/A	N/A	20 (1 studies)	**⨁⨁⨁◯ moderate**	Due to risk of bias
**Course of disease**								
acute/subacute phase	2	0.64 [-1.89, 3.18]	0.62	0	0.86	66 (2 studies)	**⨁⨁⨁⨁ high**	
chronic phase	2	0.38 [-0.97, 1.74]	0.58	66	0.08	54 (2 studies)	**⨁⨁◯◯ low**	Due to risk of bias and inconsistency
**BBS**	3	2.61 [-0.74, 5.95]	0.13	73	0.02	84 (3 studies)	**⨁⨁◯◯ low**	Due to risk of bias and inconsistency
**Number of TBS pulses**								
600 pulses	2	3.89 [-0.01, 7.78]	0.05	60	0.12	64 (2 studies)	**⨁⨁⨁◯ moderate**	Due to inconsistency
1200 pulses	1	0.60 [-1.68, 2.88]	0.61	N/A	N/A	20 (1 studies)	**⨁⨁⨁◯ moderate**	Due to risk of bias
**Course of disease**								
acute/subacute phase	1	1.58 [-2.56, 5.72]	0.45	N/A	N/A	30 (1 studies)	**⨁⨁⨁⨁ high**	
chronic phase	2	3.03 [-1.87, 7.93]	0.23	86	0.007	54 (2 studies)	**⨁◯◯◯ very low**	Due to risk of bias and inconsistency

*Abbreviations: ARAT* Action Research Arm Test, *BBS* Berg Balance Scale, *CB* Cerebellum, *CI* Confidence interval, *FMA-UE* Upper extremity Fugl-Meyer Assessment, *FMA-LE* Lower extremity Fugl-Meyer Assessment, *LBT* Line bisection test, *MAS* Modified Ashworth scale, *M1* Primary motor cortex, *NHPT* Nine-hole Peg Test, *SCT* Star cancellation test, *SMD* Standardized mean difference, *WMD* Weighted mean difference

### Cognitive impairment

Three studies with a total of 129 patients evaluated the efficacy of TBS on post-stroke cognitive impairment (PSCI) and most of them suffered a left-hemisphere stroke. The participants in two studies were in the acute phase of the stroke. All studies employed iTBS to the left dorsolateral prefrontal cortex (LH DLPFC), with a total pulse number of 600 pulses and the stimulation intensity ranging from 70 to 100% RMT. One study had a treatment period of 6 weeks, while the other two studies lasted for 2 weeks. Due to the inconsistency of outcome measures, a meta-analysis was not conducted. In terms of the results, two studies [18, 19] in stroke patients have shown that iTBS of the LH DLPFC improved global cognition with a significant improvement in executive function, and better ADL after treatment was associated with better cognitive function. Similarly, Tsai et al. found that 2-week TBS significantly improved repeatable battery for the assessment of neuropsychological status (RBANS) scores in left-hemisphere stroke patients with cognitive impairment, especially in attention and memory.

### Visuospatial neglect

Eight studies with a total of 144 patients evaluated the efficacy of TBS on post-stroke visuospatial neglect (VSN). All patients were in the acute or subacute phase of stroke, with six studies specifically enrolling patients with right-hemisphere stroke. In these studies, seven employed cTBS to the left hemisphere posterior parietal cortex (LH PPC), and one used iTBS to the LH DLPFC. The total pulse number was 600 pulses in three studies, while 801 pulses in the others. Two studies used 40% RMT in the control group, while the rest used sham control. Short-term follow-up was completed in three studies, and one study conducted a long-term follow-up. As for the meta-analysis, only the data of patients from six studies with common outcome measures (including LBT and SCT) were extracted.

#### LBT

**Fig. 4:**
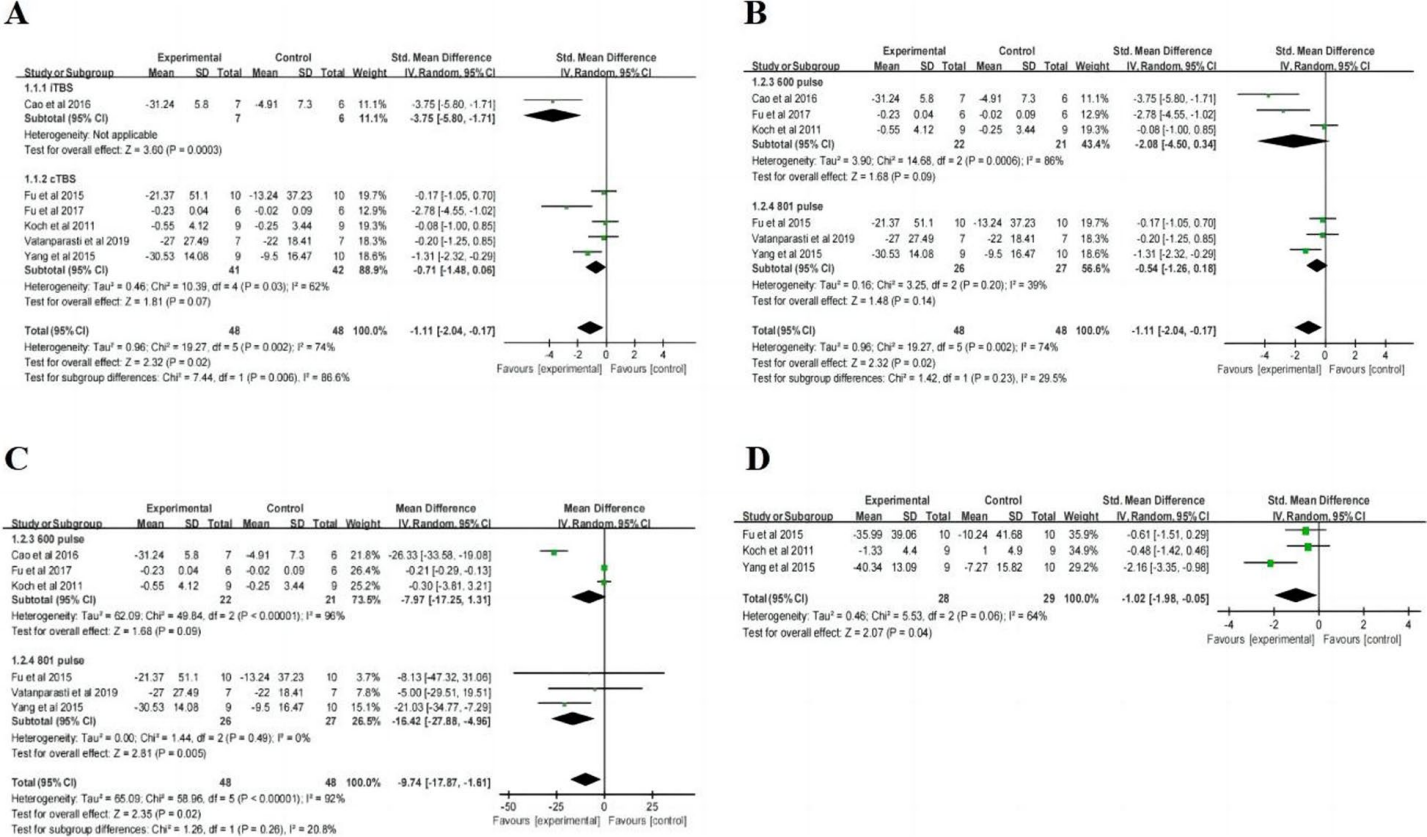
TBS on LBT scores

After the treatment, the change in LBT scores (Table 2) showed statistically significant differences between the TBS group and control group (SMD = -1.11, 95% CI = -2.04, -0.17, *P* = 0.02). Subgroup ( \* MERGEFORMAT Fig. 4A, B) analysis showed significant differences and favored the experimental group among the participants receiving iTBS (SMD = -3.75, 95% CI = -5.80, -1.71, *P* = 0.0003), but not among those receiving cTBS (SMD = -0.71, 95% CI = -1,48, 0.06, *P* = 0.07), 600 pulses (SMD = -2.08, 95% CI = -4.50, 0.34, *P* = 0.09) and 801 pulses (SMD = -0.54, 95% CI = -1.26, 0.18, *P* = 0.14). Given that the units of studies in the 801-pulse subgroup are uniform, the effect size was converted to weighted mean difference (WMD), resulting in reduced heterogeneity (*P* = 0.49, I^2^ = 0%) ( \* MERGEFORMAT Fig. 4C) and a statistically significant result (MD = -16.42, 95% CI = -27.88, -4.96, *P* = 0.005). Furthermore, TBS showed ( \* MERGEFORMAT Fig. 4D) significant improvement in LBT at short-term follow-up (SMD = -1.02, 95% CI = -1.98, -0.05, *P* = 0.04).

#### SCT

**Fig. 5:**
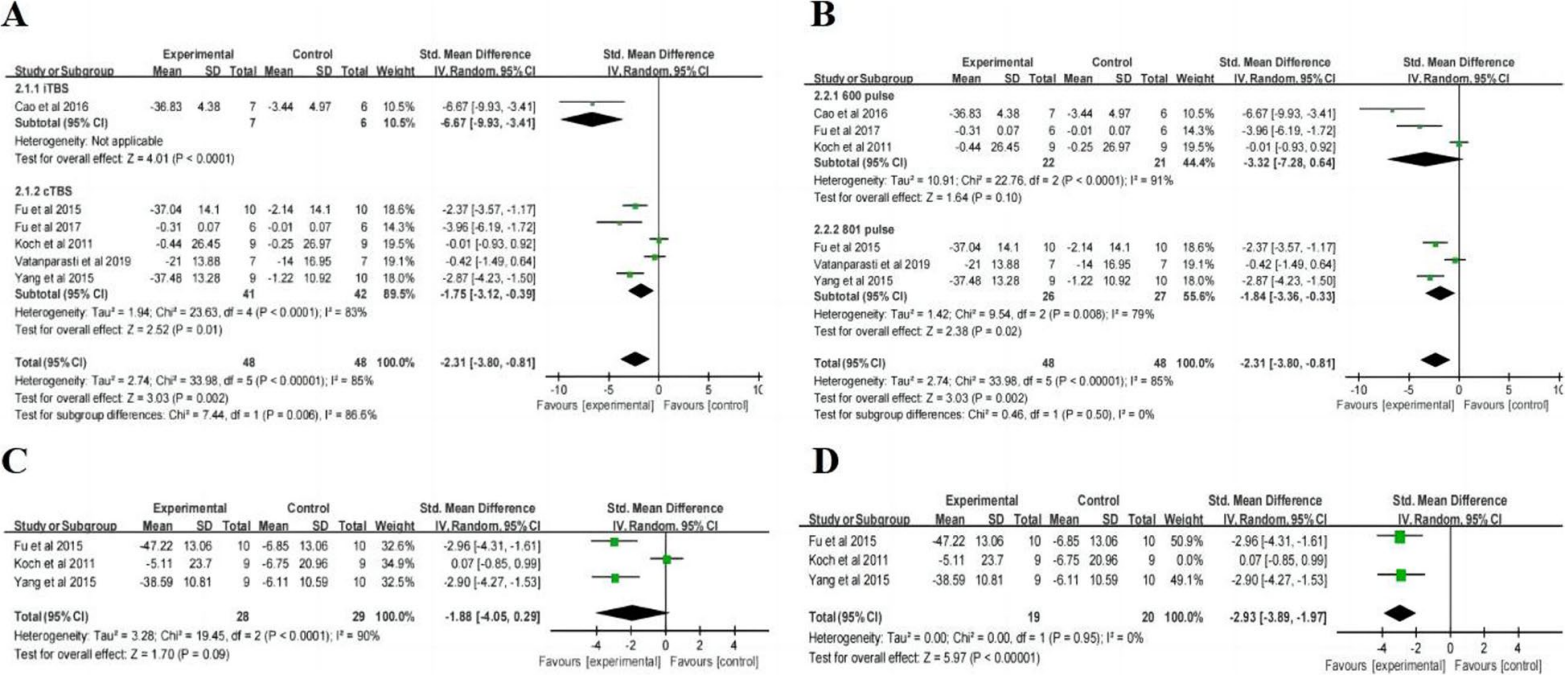
TBS on SCT scores

After the treatment, the change in SCT scores (Table 2) showed statistically significant differences between the TBS group and control group (SMD = -2.31, 95% CI = -3.80, -0.81, *P* = 0.002). Subgroup ( \* MERGEFORMAT Fig. 5A, B) analysis showed significant differences and favored the experimental group among the participants receiving iTBS (SMD = -6.67, 95% CI = -9.93, -3.41, *P* < 0.0001), cTBS (SMD = -1.75, 95% CI = -3.12, -0.39, *P* = 0.01) and 801 pulses (SMD = -1.84, 95% CI = -3.36, -0.33, *P* = 0.02), but not among those receiving 600 pulses (SMD = -3.32, 95% CI = -7.28, 0.64, *P* = 0.10). Additionally, TBS showed ( \* MERGEFORMAT Fig. 5C) no significant improvement in SCT at short-term follow-up (SMD = -1.88, 95% CI = -4.05, 0.29, *P* = 0.09). Due to the high heterogeneity of the result (*P* < 0.0001, I^2^ = 90%), sensitivity analysis was performed. After excluding the study with inconsistent units, a reanalysis ( \* MERGEFORMAT Fig. 5D) revealed decreased heterogeneity (*P* = 0.95, I^2^ = 0%) and a significant impact of TBS at short-term follow-up (SMD = -2.93, 95% CI = -3.89, -1.97, *P* < 0.00001).

Among the remaining two studies not included in the meta-analysis. Nyffeler et al. [13] found that both 8 trains and 16 trains cTBS yielded similar improvements in post-stroke VSN for up to 6 weeks. Hopfner et al. [10] observed that the effect of cTBS combined with smooth pursuit training (SPT) was superior to SPT alone on the bird cancellation task (BCT).

### Aphasia

Four studies with a total of 138 patients evaluated the efficacy of TBS on post-stroke aphasia (PSA). All studies enrolled participants with left-hemisphere stroke. Among them, three applied iTBS to the left hemisphere inferior frontal gyrus (LH IFG) in chronic patients, while one used iTBS to the right hemisphere posterior superior temporal gyrus (RH pSTG) in acute and subacute patients. The intensity of TBS was 80% RMT in two studies, and 80% AMT in the other two studies with a total pulse number of 600 pulses. One study had a treatment period of 2 weeks, while the other three studies lasted for 3 weeks. Due to the inconsistency of outcome measures, a meta-analysis was not conducted. In terms of the results, the therapeutic potential of ipsilesional iTBS in ameliorating chronic non-fluent aphasia has been supported by Chou et al. [26]. Two studies [24, 25] reported naming and semantic fluency improved immediately after iTBS treatment and persisted for at least 3 months, and the longer course of iTBS treatment had a more pronounced effect. Zheng et al. creatively employed cTBS to suppress the right STG (the homologous area to Wernicke's area) and found that the improvement in both auditory comprehension and repetition was accompanied by a significant decrease in activity in the right pars triangularis (rPTr) (the homologous area to Broca's area) and a marked increase in spontaneous neural activity in the left prefrontal cortex [23].

### Spasticity

Five studies with a total of 104 patients reported the MAS scores after TBS treatment for post-stroke spasticity (PSS) and 50% of them had a left-hemisphere stroke. The patients in three studies were in the chronic phase, while the other two were in the acute or subacute phase. In these studies, three applied iTBS to the affected hemisphere primary motor cortex (AH M1), one applied iTBS to the ipsilesional cerebellum (CB) and one applied cTBS to the unaffected hemisphere primary motor cortex (UH M1). The stimulation intensity was 110% RMT in one study, and 80% AMT in the other four studies, with a total pulse number of 600 pulses over 2 weeks. only one study completed a short-term follow-up.

**Fig. 6:**
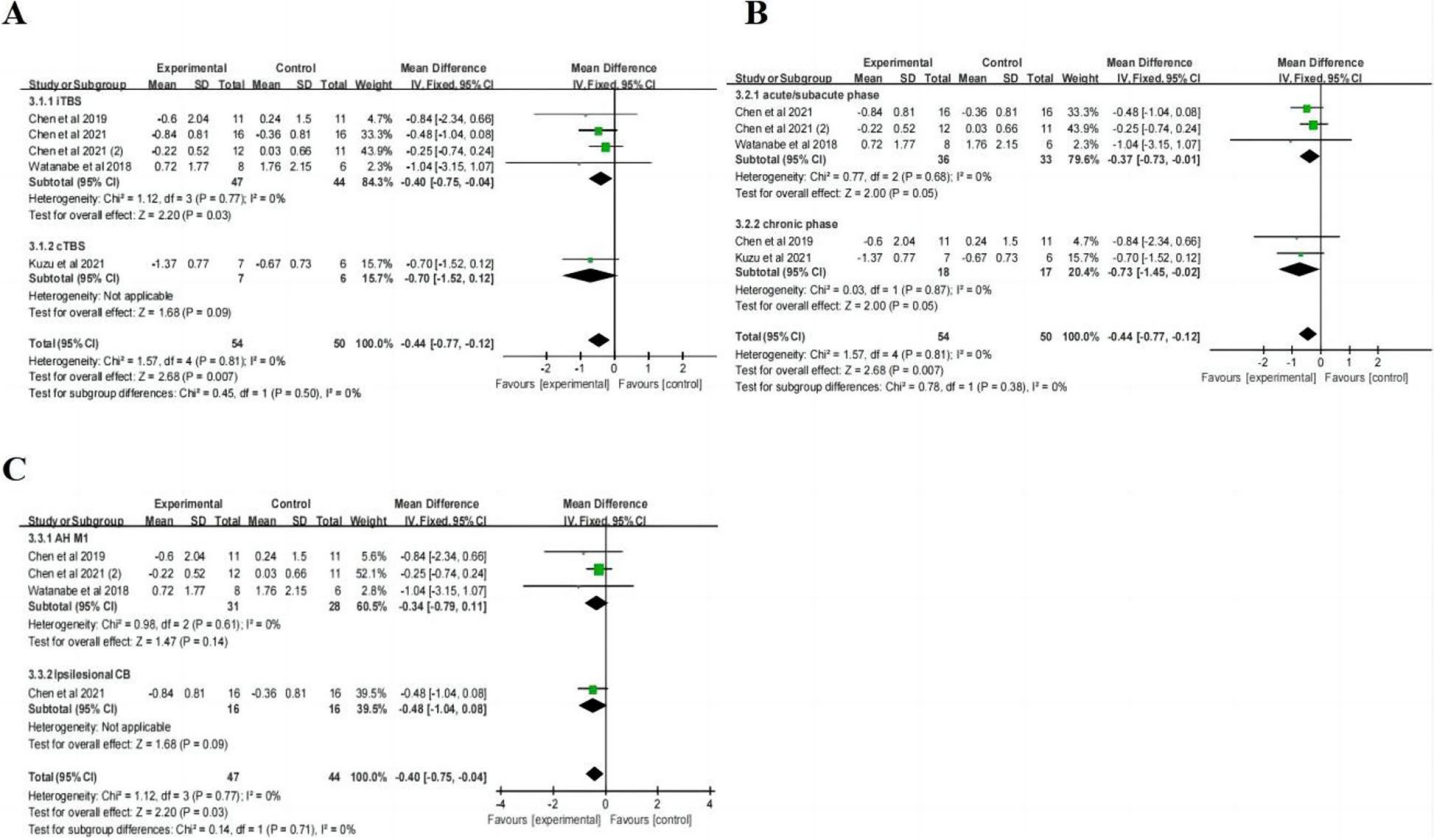
TBS on MAS scores

Meta-analysis (Table 2) revealed that the change in MAS scores was statistically significant after TBS treatment compared to the control group (MD = -0.44, 95% CI: -0.77, 0.12, *P* = 0.007). Subgroup analysis ( \* MERGEFORMAT Fig. 6A, B) showed significant effect sizes for recovery of PSS in acute/subacute phase (MD = -0.37, 95% CI: -0.73, -0.01,* P* = 0.05), chronic phase (MD = -0.70, 95% CI: -1.52, 0.12, *P* = 0.05), iTBS (MD = -0.40, 95% CI: -0.75, -0.04, *P* = 0.03), but not cTBS (MD = -0.70, 95% CI: -1.52, 0.12, *P* = 0.09). Furthermore, subgroup analysis ( \* MERGEFORMAT Fig. 6C) of the targeted area revealed that there was no statistically significant effect of iTBS on AH M1 (MD = -0.34, 95% CI: -0.79, 0.11, *P* = 0.14) and ipsilesional CB (MD = 0.48, 95% CI: -1.04, 0.08, *P* = 0.09).

### Hand

Four studies with a total of 111 patients reported the NHPT scores after TBS treatment for post-stroke finger dexterity and 42% of them had a left-hemisphere stroke. Two studies included patients in the subacute phase of stroke, and the other two studies specifically enrolled patients in the chronic phase of ischemic stroke. One study [38] applied two types of TBS protocols and included separate control groups for each, hence we treated them as two independent experiments for analysis. Therefore, among these studies, two applied iTBS to the AH M1, two applied cTBS to the UH M1, and one applied cTBS to the AH M1. The stimulation intensity was 70% RMT in one study, and 80% AMT in the other four studies, with a total pulse number of 600 pulses over 2–3 weeks. Short-term follow-up was completed in one study, while two studies completed both 1 month and 3 months of follow-up.

**Fig. 7:**
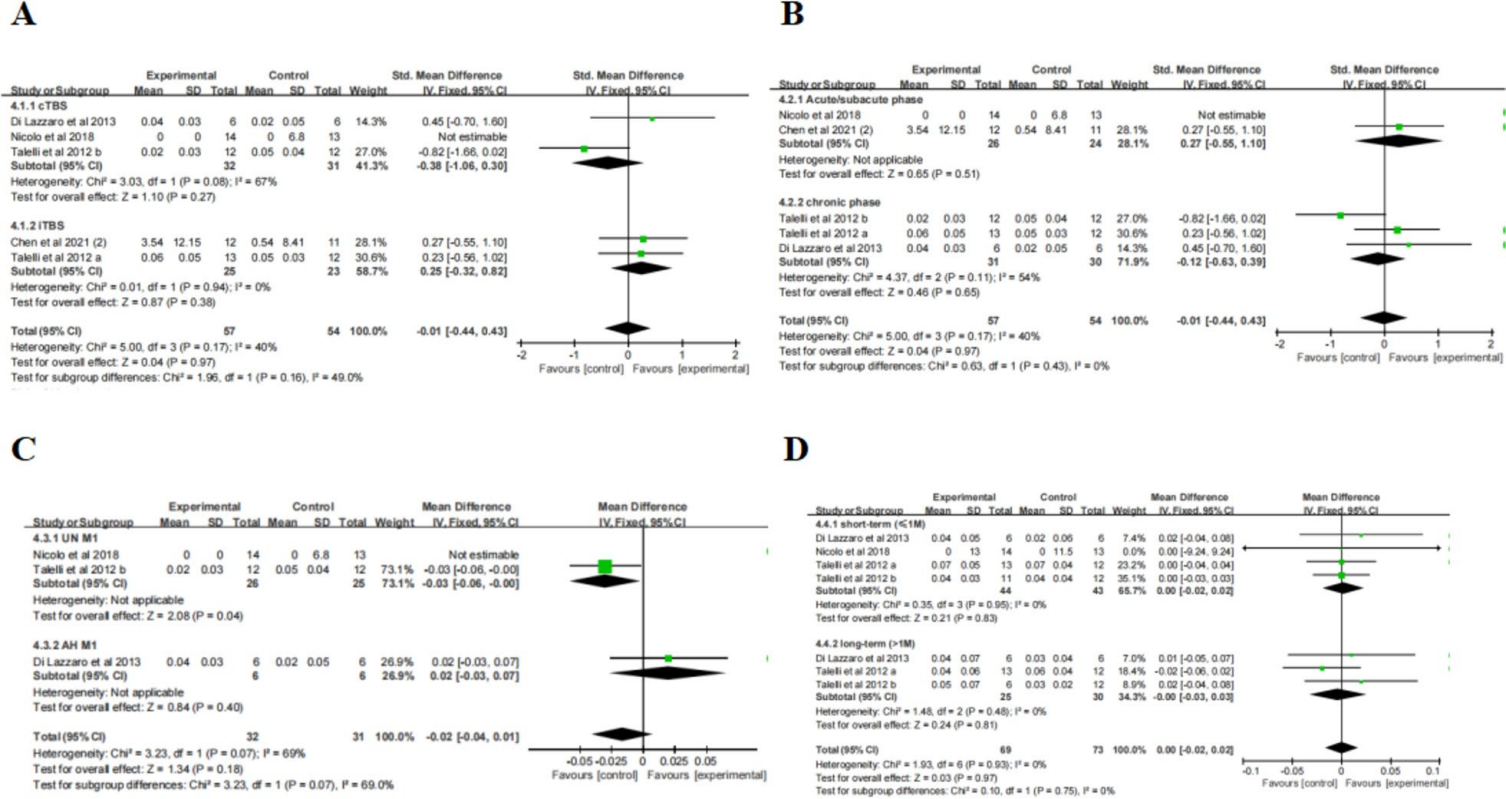
TBS on NHPT scores

Meta-analysis (Table 2) revealed that the change in NHPT scores ( \* MERGEFORMAT Fig. 7A) was not statistically significant after TBS treatment compared to the control group (SMD = -0.01, 95% CI = -0.44, 0.43, *P* = 0.97). Subgroup analysis showed no significant differences in terms of type of stimulation, course of disease, and follow-up ( \* MERGEFORMAT Fig. 7A, B, D). Moreover, in one study, the results ( \* MERGEFORMAT Fig. 7C) showed that the control group had better outcomes compared to individuals receiving cTBS over UH M1(MD = -0.03, 95% CI = -0.06, -0.00, *P* = 0.04).

### Upper extremity

Ten studies with a total of 237 patients evaluated the efficacy of TBS on post-stroke upper limb motor function, including eight reported the results of the upper extremity Fugl-Meyer Assessment (FMA-UE) and five reported the results of the ARAT. Except for one study that did not report, 47% of the patients had a left-hemisphere stroke. Among these studies, seven recruited participants in the chronic phase, two in the subacute phase, and one in the acute phase. Six studies applied iTBS to the AH M1, two applied cTBS to the UH M1, one applied cTBS to the AH M1, and one combined iTBS and cTBS. In one study [36], besides iTBS, the experimental group also applied priming iTBS (cTBS stimulation before iTBS treatment), thus we divided it into two groups for analysis. The stimulation intensity was 80%AMT in most of the included studies, with a total pulse number of 600 pulses over 2–4 weeks. Short-term follow-up was completed in three studies, while two studies completed 1 month and 3 months of follow-up and one study completed 3 months, 6 months, and 1 year of follow-up.

#### FMA-UE

**Fig. 8:**
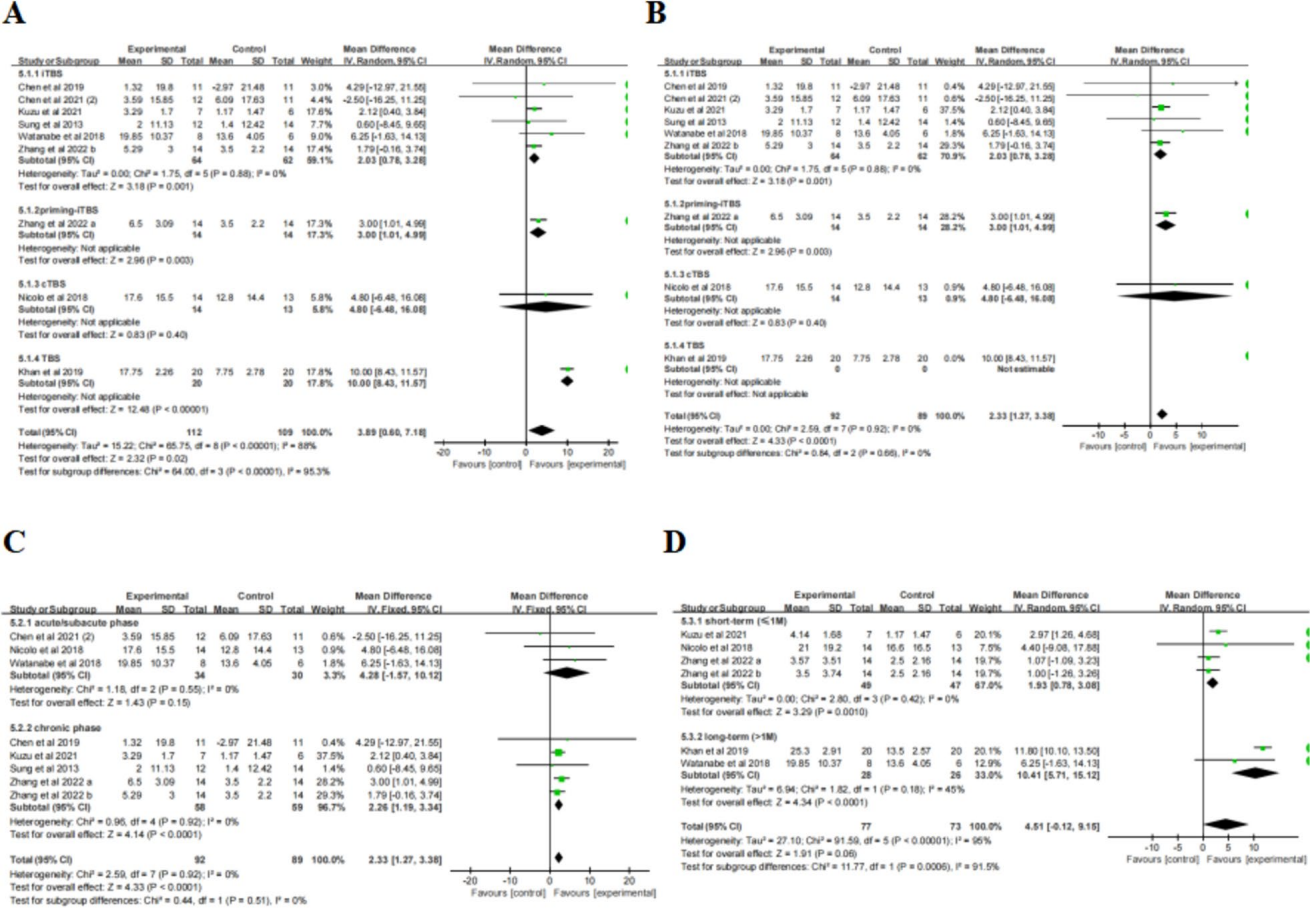
TBS on FMA-UE scores

Meta-analysis (Table 2) revealed that the change in FMA-UE scores was statistically significant after TBS treatment compared to the control group (MD = 3.89, 95% CI = 0.60, 7.18,* P* = 0.02). Subgroup analysis ( \* MERGEFORMAT Fig. 8A) showed significant differences and favored the experimental group among the participants receiving iTBS (MD = 2.03, 95% CI = 0.78, 3.28, *P* = 0.001), priming-iTBS (MD = 3.00, 95% CI = 1.01, 4.99, *P* = 0.003) and combined TBS (MD = 10.00, 95% CI = 8.43, 11.57, *P* < 0.00001), but not among those receiving cTBS (MD = 4.80, 95% CI = -6.48, 16.08, *P* = 0.40). Due to the high heterogeneity of the result (*P* < 0.0001, I^2^ = 90%), sensitivity analysis was performed. After excluding one study, a reanalysis ( \* MERGEFORMAT Fig. 8B) revealed decreased heterogeneity (*P* = 0.92, I^2^ = 0%), and the results were consistent. Further subgroup analysis ( \* MERGEFORMAT Fig. 8C) showed significant effect sizes only in the chronic phase (MD = 2.26, 95% CI = 1.19, 3.34, *P* < 0.0001). Moreover, TBS showed ( \* MERGEFORMAT Fig. 8D) significant improvement in FMA-UE at short-term follow-up (MD = 1.93, 95% CI = 0.78, 3.08, *P* = 0.001) and long-term follow-up (MD = 10.41, 95% CI = 5.71, 15.12, *P* < 0.0001).

#### ARAT

**Fig. 9:**
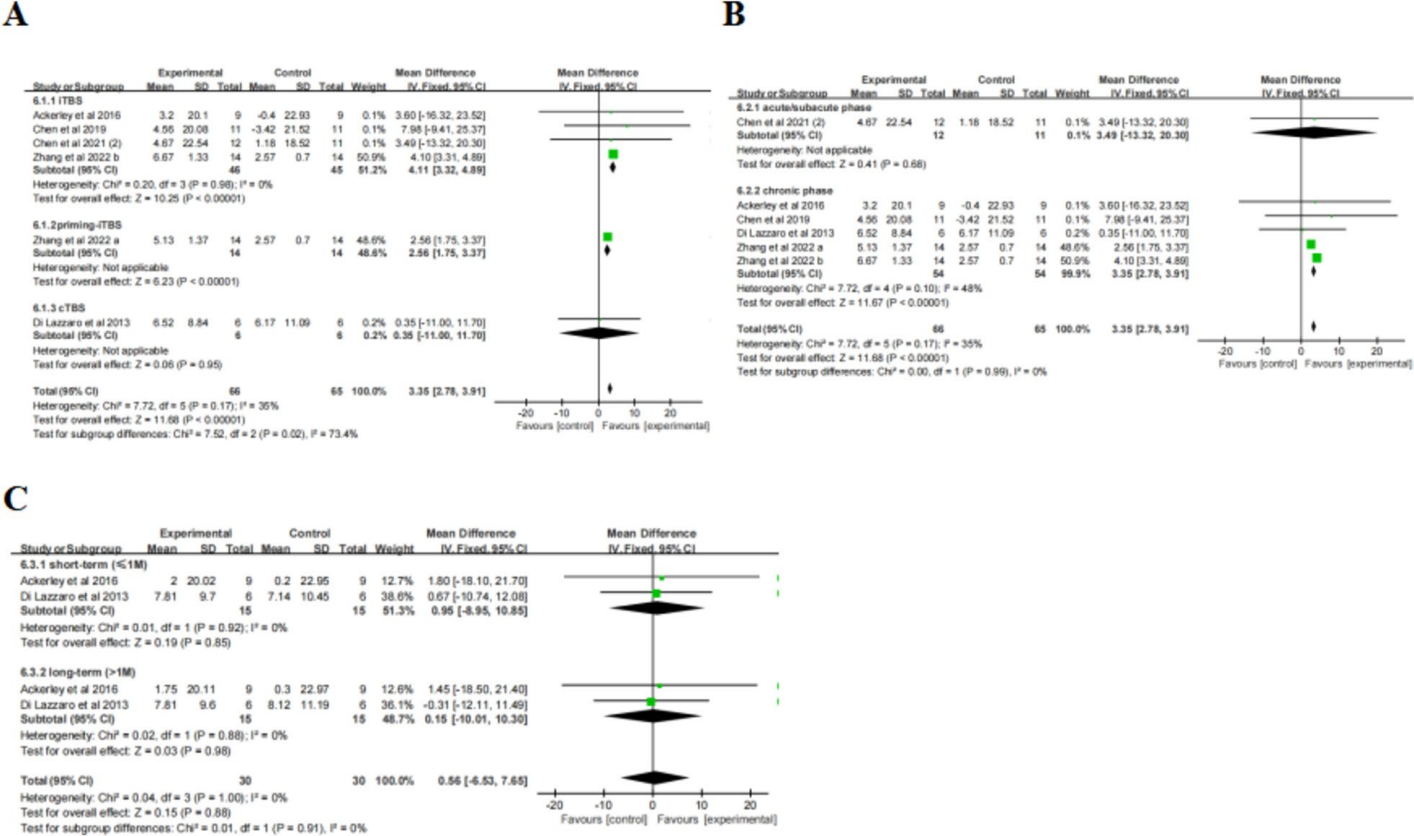
TBS on ARAT scores

Meta-analysis (Table 2) revealed that the change in ARAT scores was statistically significant after TBS treatment compared to the control group (MD = 3.35, 95% CI = 2.78, 3.91, *P* < 0.00001). Subgroup analysis ( \* MERGEFORMAT Fig. 9A) showed significant differences and favored the experimental group among the participants receiving iTBS (MD = 4.41, 95% CI = 3.32, 4.89, *P* < 0.00001), and priming-iTBS (MD = 2.56, 95% CI = 1.75, 3.37, *P* < 0.00001), but not among those receiving cTBS (MD = 0.35, 95% CI = -11.00, 11.70, *P* = 0.95). Further subgroup analysis ( \* MERGEFORMAT Fig. 9B) showed significant effect sizes only in the chronic phase (MD = 3.35, 95% CI = 2.78, 3.91, *P* < 0.00001). However, TBS showed ( \* MERGEFORMAT Fig. 9C) no significant improvement in ARAT at short-term follow-up (MD = 0.95, 95% CI = -8.95, 10.85, *P* = 0.85) or long-term follow-up (MD = 0.15, 95% CI = -10.01, 10.30, *P* = 0.98).

### Lower *extremity*/balance

Four studies with a total of 120 patients evaluated the efficacy of TBS on post-stroke lower limb motor function and balance, including four reported the results of the lower extremity Fugl-Meyer Assessment (FMA-LE) and three reported the results of the BBS. 48% of the patients had a left-hemisphere stroke. The patients in two studies were in the chronic phase, while the other two were in the acute or subacute phase. Among these studies, two applied iTBS to the ipsilesional CB, one applied iTBS to the bilateral M1, and one applied iTBS to the contralesional CB. The stimulation intensity in three studies was set at 80% AMT, with a total pulse number of 600 pulses over 2–3 weeks. One study used 1200 pulses in total, 100% midline MT, 2 session per week over 5 weeks.

#### FMA-LE

**Fig. 10:**
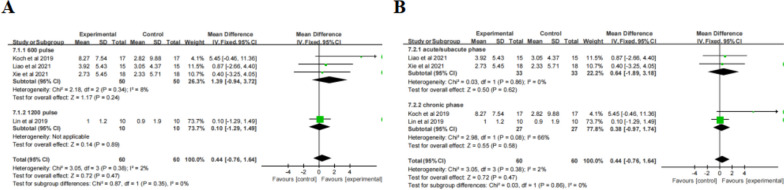
TBS on FMA-LE scores

Meta-analysis (Table 2) revealed that the change in FMA-LE scores was not statistically significant after iTBS treatment compared to the control group (MD = 0.44, 95% CI = -0.76, 1.64,* P* = 0.47). Accordingly, subgroup analysis ( \* MERGEFORMAT Fig. 10A, B) didn’t show significant effect sizes in terms of TBS pulses or course of disease.

#### BBS

**Fig. 11:**
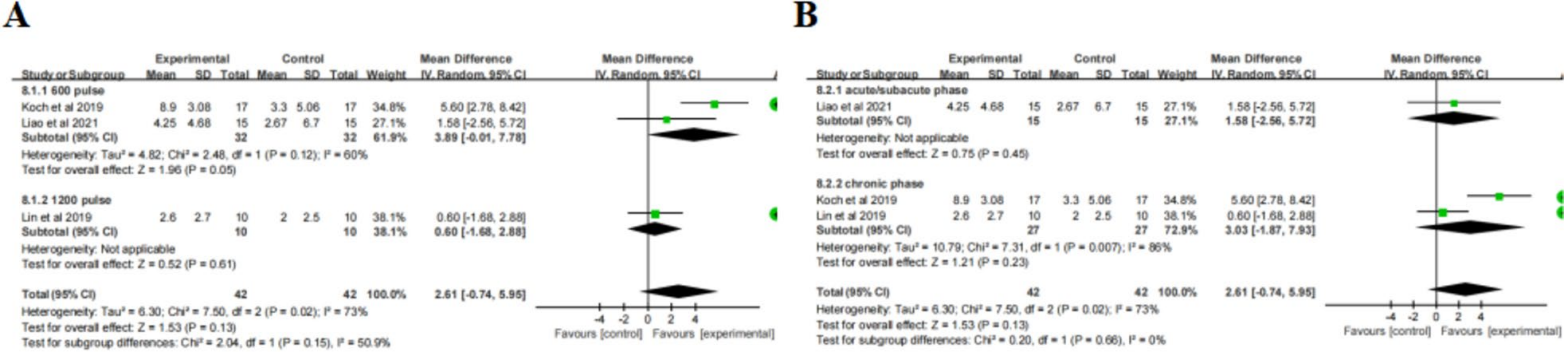
TBS on BBS scores

Meta-analysis (Table 2) revealed that the change of BBS scores was not statistically significant after iTBS treatment compared to control group (MD = 2.61, 95% CI = -0.74, 5.95,* P* = 0.13) both ( \* MERGEFORMAT Fig. 11B) in the acute/subacute phase (MD = 1.58, 95% CI = -2.56, 5.72, *P* = 0.45) or the chronic phase (MD = 3.03, 95% CI = -1.87, 7.93, *P* = 0.23). Subgroup analysis ( \* MERGEFORMAT Fig. 11A) showed that the effect of 600 pulses was significant (MD = 3.89, 95% CI = -0.01, 7.78, *P* = 0.05), whereas 1200 pulses was not (MD = 0.60, 95% CI = -1.68, 2.88, *P* = 0.61). It is worth noting that 600-pulse stimulations targeted to ipsilesional CB, while 1200-pulse targeted to bilateral M1.

### Dysphagia

Two studies with a total of 102 patients reported the effects of iTBS for post-stroke dysphagia (PSD) in the acute or subacute phase and 42% of them had a left-hemisphere stroke. Both of them applied iTBS with a total pulse number of 600 pulses over 2 weeks. Due to the inconsistency of outcome measures, a meta-analysis was not conducted. In terms of the results, Xie et al. [43] applied iTBS to the swallowing motor cortex of the affected hemisphere, the results of the iTBS group showed that the improvement in the Penetration/Aspiration Scale (PAS) scores at 2 weeks and the water-swallowing test (WST) and Murray Secretion Scale (MSS) scores at 4-week follow-up was significantly greater than that in the sham stimulation group. Interestingly, Rao et al. [44] applied iTBS to the bilateral CB, an efficient improvement in swallowing function after the 2-week treatment and at the 4-week follow-up.

## Discussion

In this study, we assessed the efficacy of TBS on various functional impairments in stroke patients. On the whole, the majority of the results support the positive effects of TBS. Theoretically, the ipsilesional iTBS and contralesional cTBS protocols have been widely used based on the IHI model [14], especially in the motor system. However, the protocol that dominant-lateral stimulation and contralateral inhibition are commonly used in patients with cognitive impairment characterized by hemispheric lateralization including VSN associated with right hemisphere dominance as well as PSCI and PSA associated with left hemisphere dominance. In detail, the limited available data support the role of iTBS over the LH DLPFC for treating PSCI, especially the executive function, memory, and attention, which is consistent with a meta-analysis that has reported the effectiveness of conventional TMS for PSCI [45]. Moreover, Tsai et al. [17] have suggested that iTBS was less effective than 5 Hz rTMS in enhancing attention, but equally effective in improving overall cognitive and memory function. The electroencephalogram (EEG) indicated differences in high- or low-frequency band power between two stimulation methods may correspond to dissimilar modulating effects.

For VSN, the current evidence supports the immediate and short-term after-effect of cTBS over LH PPC in acute/subacute patients. But the quality of the evidence is low due to risk of bias and inconsistency. It is worth noting that the modified cTBS protocol (801 pulses) seems to be more effective compared to standard 600-pulse stimulation. Our result is in part consistent with another two meta-analyses [46, 47], unfortunately, which failed to address the potential variations in therapeutic efficacy among different pulse numbers of TBS intervention. It is notable that one of them [46] suggested that TBS was more effective than other noninvasive brain stimulation protocols. In particular, iTBS over the LH DLPFC is also effective for VSN, and resting-state functional magnetic resonance imaging (fMRI) showed that the functional connectivity was significantly reduced in the right attention network. Besides, Yang et al. [9] found that cTBS was superior to 1 Hz and 10 Hz rTMS on behavioral scores and exhibited a significant increase in fractional anisotropy (FA) of the left external capsule as observed by diffusion tensor imaging (DTI).

For PSA, iTBS over the homologous area of Broca’s or Wernicke’s region in the dominant hemisphere and cTBS over the contralateral hemisphere have been shown to enhance language abilities in subacute and chronic patients, including naming, comprehension, fluency, and repetition. Importantly, the therapeutic effects have been demonstrated to be maintained for up to 3 months. Similar conclusions have been drawn in previous studies [48, 49] for LF-rTMS. However, iTBS was proved by Chou et al. [26] to improve auditory comprehension over 1 Hz rTMS. On the one hand, the non-dominant right hemisphere may have inherently lower proficiency in language processing compared to the dominant left hemisphere. On the other hand, they suggested that LF-rTMS might be more beneficial in the subacute phase of stroke, whereas HF-rTMS might be more suitable for chronic patients.

For PSS, Xu et al. [50] published a meta-analysis that no significant reduction of rTMS (including iTBS) in MAS scores, only two of the five articles included in the analysis used iTBS, one of which showed no improvement in spasticity with small sample size. In our study, moderate-quality evidence supports a beneficial effect of iTBS on PSS and has been shown in both AH M1 and ipsilesional cerebellum. Besides, 1 Hz rTMS combined with cerebellar cTBS exhibited better efficacy than each of them alone in treating PSS and limb dyskinesia, but no significant difference was found between 1 Hz rTMS and cTBS [51]. In the study conducted by Kuzu et al. [27], cTBS did not show any benefits compared to 1 Hz rTMS in terms of pronator and finger flexor spasticity, and the only observed after-effect was in wrist flexor spasticity at a 4-week follow-up.

For motor function, the results demonstrated that targeting M1 with TBS is ineffective when assessing fine motor and manual skills using the NHPT. Interestingly, a small exploratory study [37] tested the idea of applying cTBS to the stroke hemisphere and reported a significant improvement in the Jebsen-Taylor Test (JTT). The results from both FMA-UE and ARAT indicated that iTBS over the AH M1 was effective for upper limb function recovery in chronic patients, but short-term and long-term after-effects were only observed in FMA-UE scores. Besides, standard 600-pulse stimulation showed a better effect on motor function improvement compared to 1200 pulses. The results are consistent with previous studies [16, 52, 53]. Additionally, two studies [34] integrated different forms of rTMS and reported that bi-hemispheric stimulation (1 Hz rTMS to the UH M1 and iTBS to the AH M1) was associated with better motor performance when compared to unilateral modulation [54]. When comparing the effects of rTMS and TBS on hand and upper limb function in stroke patients, Watanabe et al. [31] reported that contralesional 1-Hz rTMS decreased the spasticity of the affected limb and ipsilesional iTBS improved the movement of the affected limb. Chen et al. [55] summarized the effect of rTMS on the upper limb and fine motor function during various phases of stroke, and found that TBS was more effective than rTMS in the acute phase of stroke, while the opposite was true in subacute and chronic phase. Similarly, Xia et al. [56] conducted a network meta-analysis that suggested that iTBS might be the preferred option for patients within one month from onset, whereas ≥ 10 Hz rTMS for mild stroke, severe stroke, and the convalescent phase. However, results should be interpreted with caution due to the relatively small sample sizes in some subgroups. Unfortunately, our study did not find any benefits of TBS in the recovery of lower limb motor function after stroke, only 600-pulse iTBS showed a certain therapeutic effect in improving balance.

For PSD, limited research suggested that iTBS to the affected suprahyoid motor cortex or bilateral cerebellum might be effective. Yu-Lei et al.[57] argued that iTBS exerted similar efficacy, safety, and tolerability compared to 10 Hz rTMS.

### Pathophysiological mechanism

The brain would go through several recovery phases after stroke, spontaneously reorganizing neural circuits and producing neuroplastic phenomena. Neuroplasticity was suggested as the rationale for using TBS in stroke recovery [58]. At the molecular level, TBS could adjust synaptic efficacy in glutamatergic and gamma-aminobutyric acid (GABA)-mediated circuits, inducing long-term potentiation (LTP)-like or long-term depression(LTD)-like plasticity[59]. Correspondingly, various studies have demonstrated that functional improvement after several days/weeks of TBS treatment in stroke patients can persist for a short duration of 2 weeks to as long as 2 years [9, 21, 22, 24, 25, 27, 32, 44]. At the network level, human and animal research both have shown a decline in resting state functional connectivity(RSFC) of cerebral networks after stroke, and TBS yields the ability to reverse the decline in intra- and inter-hemispheric connectivity of cerebral networks [60, 61]. Furthermore, the effect of TBS is not limited to surrounding regions but extends to other neural networks, that is, from local punctate activation at the stimulated site to flake activation [19].

### Influencing factors

Technical factors, such as the number of pulses, intensity, and duration of stimulation, may play a crucial role in predicting TBS outcomes. Based on the mechanisms of LTD/LTP [62], the effect of TBS should be dose-dependent at the local level (cortical excitability) and systemic systems level (functional connectivity). In this regard, repeated trains [11, 63], higher intensity [20, 22], and longer duration of stimulation [25] could enhance and prolong the efficacy of TBS. For instance, Yang et al. [9] found that patients responded best at 1 month after the end of treatment. Besides, the potentially cumulative physiological effects of bilateral stimulation [34, 35, 54] and paired target stimulation [64] might be more significant than unilateral stimulation or single target stimulation for spasticity and motor function recovery.

When trying to achieve a more significant effect with larger doses of TBS, it's necessary to introduce a new concept of metaplasticity. The term “meta” reflects higher-order plasticity, known as synaptic plasticity [65]. Metaplasticity can be described as a homeostatic synaptic plasticity with the characteristic of negative feedback to prevent over- and under-excitability in neural networks [66]. A small exploratory study [37] applied cTBS to the stroke hemisphere, revealing that ipsilesional cTBS is safe and may enhance the response to conventional therapy through a steady increase in learning ability. Zhang et al.’s study indicated that priming iTBS produced a more stable after-effect compared to non-priming iTBS [36]. EEG showed that priming iTBS had an advantage in enhancing the high β-event-related desynchronization induced by mirror visual feedback, suggesting that the variability of the facilitatory response induced by iTBS after cTBS initiation was reduced. Similarly, ipsilesional cTBS before physical therapy has the potential to enhance better relearning by inducing LTD-like effects on the stroke hemisphere [37], which again confirmed that opposite priming effects promote the regulation of metaplasticity in a homeostatic manner. However, several studies have shown that doubling the trains [13] or pulses [41] of TBS, cannot enhance or even reverse TBS-induced plasticity. These findings suggest that a sufficient dose of a specific TBS protocol would ‘stabilize and lock’ the cortical excitability at an optimal level, which reversely would be inhibited when excessive doses are applied.

In addition, although our study did not specifically analyze it, the differences in lesion location and nerve injury degree are likely to influence the TBS effect in stroke patients. A previous study [67] has shown that patients with subcortical lesions show greater improvement after rTMS than those with cortical lesions. Besides, functional improvement may be limited in patients with mild to moderate severity, which may be related to “the ceiling effect”. Precisely, based on the bimodal balance recovery model, the structural reserve is sufficient to respond to TBS in patients with below-threshold damage, while no response when the damage is extremely severe above the threshold [68].

### Limitations

Several limitations of this meta-analysis should be noted when interpreting the results. First, most of the included studies had small sample sizes and varied in both the parameter and duration of intervention. Although subgroup analyses were performed, there was still high heterogeneity in some of the results. Second, the forms of the adjuvant treatments during TBS also varied across the studies. Third, the diversity of assessment tools and outcome measures’ units limits the availability of analyzable data and may potentially lead to deviations. Fourth, we only considered studies published in English, raising the possibility of bias if relevant studies have been released in other languages. Finally, it remains unclear whether factors such as age, severity of the injury, type of injury, and adjuvant therapy have an impact on the outcomes of TBS. Further research is needed to explore their potential influence.

## Conclusion, future directions

Though TBS is not the first-line treatment in stroke rehabilitation, it plays an important role in ameliorating symptoms and augmenting the efficacy of other conventional rehabilitative methods. This meta-analysis further summarizes the role of TBS therapy for post-stroke dysfunctions, including iTBS over the LH DLPFC for PSCI, the modified cTBS over the LH PPC for VSN in the acute/subacute phase, iTBS over the LH IFG and cTBS over the RH pSTG for PSA in subacute and chronic phase, iTBS over the AH M1 or CB for PSS, tandard 600-pulse iTBS over the AH M1 for upper limb function in chronic phase and last for 3 months, and standard 600-pulse iTBS for balance and PSD. In addition, more pulses and higher intensity of stimulation within a certain range may lead to significant effects, and bilateral stimulation, paired target stimulation, and priming iTBS have all been shown to enhance benefits in the field of motor rehabilitation. While there is no clear evidence indicating that TBS is superior to TMS, TBS may be a potential alternative to traditional rTMS in terms of increasing capacity, improving efficiency, and shorting waiting time.

Given the limited number of current studies and their heterogeneity, there is still controversy regarding the efficacy and underlying mechanism of TBS. Future trials should incorporate electrophysiological methods and advanced multimodal imaging techniques to determine the optimal technical settings and intervention timing for stroke survivors.

### Supplementary Information

**Additional file 1.**

**Additional file 2.**

**Additional file 3.**

## Data Availability

All data generated or analysed during this study are included in this published article and its supplementary information files.
