# Supplementary material for: Theta burst stimulation: what role does it play in stroke rehabilitation? A systematic review of the existing evidence
Source: BMC Neurol. 2024 Feb 1;24:52. doi: 10.1186/s12883-023-03492-0 (PMC10832248; doi:10.1186/s12883-023-03492-0)
Supplement: Supplementary file 1 — Additional file 1. [file 12883_2023_3492_MOESM1_ESM.docx]

**Search queries and results**

**Pubmed search strategy:**

((stroke[MeSH Terms]) OR (cerebrovascular accident[MeSH Terms])) AND ((((theta burst stimulation[Title/Abstract]) OR (TBS[Title/Abstract])) OR (cTBS[Title/Abstract])) OR (iTBS[Title/Abstract]) AND ((clinicaltrial[Filter] OR randomizedcontrolledtrial[Filter]) AND (humans[Filter])))

Result:42

**Ovid Medline search strategy:**

((Theta burst stimul* or TBS) and (Stroke or Cerebrovascular Accident)).mp.

limit to (english language and humans)

Result:129

**Cochrane library search strategy:**

TBS in Title Abstract Keyword AND stroke in Title Abstract Keyword AND "theta burst stimul*" in Title Abstract Keyword AND cerebrovascular accident in Title Abstract Keyword Search limits: in Trials (Word variations have been searched)

Result:254

**Embase search strategy:**

((Theta burst stimul* or TBS) and (Stroke or Cerebrovascular Accident)).mp.

limit to (human and english language)

limit to (article)

Result:155

**Web of Science search strategy:**

((TS=(cerebrovascular accident)) OR TS=(Stroke)) AND TS=("theta burst stimulation" OR " TBS " )

refine: Clinical Trial (Document Types) and English (Languages) and Humans (MeSH Headings)

Result:74
