# Supplementary material for: Theta burst stimulation: what role does it play in stroke rehabilitation? A systematic review of the existing evidence
Source: BMC Neurol. 2024 Feb 1;24:52. doi: 10.1186/s12883-023-03492-0 (PMC10832248; doi:10.1186/s12883-023-03492-0)
Supplement: Supplementary file 2 — Additional file 2. [file 12883_2023_3492_MOESM2_ESM.docx]

**List of abbreviations**

affected hemisphere (AH)

active motor threshold (AMT)

Action Research Arm Test (ARAT)

Berg Balance Scale (BBS)

cerebellum (CB)

continuous TBS (cTBS)

dorsolateral prefrontal cortex (DLPFC)

diffusion tensor imaging (DTI)

electroencephalogram (EEG)

Fugl-Meyer Assessment (FMA)

functional magnetic resonance imaging (fMRI)

inferior frontal gyrus (IFG)

intermittent TBS (iTBS)

interhemispheric inhibition (IHI)

line bisection test (LBT)

lower extremity (LE)

left-hemisphere (LH)

long-term potentiation (LTP)

long-term depression(LTD)

Modified Ashworth scale(MAS)

primary motor cortex (M1)

Nine-hole Peg Test (NHPT)

posterior parietal cortex (PPC)

post-stroke aphasia (PSA)

post-stroke cognitive impairment (PSCI)

post-stroke dysphagia (PSD)

posterior superior temporal gyrus (pSTG)

randomized controlled trials (RCTs)

right-hemisphere (RH)

resting motor threshold (RMT)

repetitive transcranial magnetic stimulation (rTMS)

star cancellation test (SCT)

theta burst stimulation (TBS)

right pars triangularis (rPTr)

standard mean difference (SMD)

upper extremity (UE)

unaffected hemisphere (UH)

visuospatial neglect (VSN)

weighted mean difference (WMD)
